# Supplementary material for: Single-molecule digital sizing of proteins in solution
Source: Nat Commun. 2024 Sep 4;15:7740. doi: 10.1038/s41467-024-50825-9 (PMC11375031; doi:10.1038/s41467-024-50825-9)
Supplement: Supplementary file 1 — Supplementary Information [file 41467_2024_50825_MOESM1_ESM.pdf]

Supplementary Information for

Single-molecule digital sizing of proteins in solution

Georg Krainer,[1,2,#,*] Raphael P.B. Jacquat,[2,#] Matthias M. Schneider,[2,8,#] Timothy J. Welsh,[2,#] Jieyuan Fan,[2,3,#] Quentin A. E. Peter,[2] Ewa A. Andrzejewska,[2] Greta Šneiderienė,[2] Magdalena A. Czekalska,[2] Hannes Ausserwoeger,[2] Lin Chai,[2] William E. Arter,[2] Kadi L. Saar,[2] Therese W. Herling,[2] Titus M. Franzmann,[4] Vasilis Kosmoliaptsis,[5,6,7] Simon Alberti,[4] F. Ulrich Hartl,[8,9] Steven F. Lee,[3] Tuomas P.J. Knowles[2,10,*]

[1] Institute of Molecular Biosciences (IMB), University of Graz, Humboldtstraße 50, 8010 Graz, Austria

[2] Centre for Misfolding Diseases, Yusuf Hamied Department of Chemistry, University of Cambridge, Lensfield Road, Cambridge CB2 1EW, UK

[3] Yusuf Hamied Department of Chemistry, University of Cambridge, Lensfield Road, Cambridge CB2 1EW, UK

[4] Center for Molecular and Cellular Bioengineering, Biotechnology Center, Technische Universität Dresden, Tatzberg 47/49, 01307 Dresden, Germany

[5] Department of Surgery, University of Cambridge, Addenbrooke's Hospital, Hills Road, Cambridge CB2 0QQ, UK

[6] NIHR Blood and Transplant Research Unit in Organ Donation and Transplantation, University of Cambridge, Hills Road, Cambridge CB2 0QQ, UK

[7] NIHR Cambridge Biomedical Research Centre, Hills Road, Cambridge CB2 0QQ, UK

[8] Department of Cellular Biochemistry, Max-Planck Institute of Biochemistry, Am Klopferspitz 18, 82152 Martinsried, Germany

[9] Munich Cluster for Systems Neurology (SyNergy), Feodor-Lynen-Str. 17, 81377 Munich, Germany

[10] Cavendish Laboratory, Department of Physics, University of Cambridge, JJ Thomson Ave, Cambridge CB3 0HE, UK

[#] Contributed equally to this work

* Correspondence should be address to: georg.krainer@uni-graz.at and tpjk2@cam.ac.uk

Supplementary Note 1

Analysis of diffusion profiles using the advection–diffusion model

Overview. To extract the hydrodynamic radius R_H, we analyze the experimental diffusion profiles using an advection–diffusion model. This involves fitting the experimental profiles with a sequence of simulated profiles, referred to as basis functions, generated through numerical simulations. By applying a least-squares error algorithm, we identify the simulated profiles that best corresponds to the experimental data, which enables us to extract the diffusion coefficient D. Subsequently, R_H is deduced using the Stokes–Einstein equation.

Generation of basis functions. The basis functions are generated following a method described by Q.A.E. Peter.[1] These functions are derived from a dimensionless differential equation rooted in the advection–diffusion equation, which describes the diffusion of species in a Poiseuille flow within a rectangular microfluidic channel. The model considers the diffusion of particles in a steady-state flow with a constant flow rate Q within a channel of height H and width W.

Basis functions are derived by first considering the general advection–diffusion equation for the local concentration $c(x, z, y)$ of the species within the channel. This equation accounts for the combined effects of advection due to fluid flow and molecular diffusion. The advection–diffusion equation is given by:

$$\frac{\partial c}{\partial t} = \nabla \cdot (D\nabla c) - \nabla \cdot (\vec{v}c) \qquad \text{(S1)}$$

with D being the diffusion coefficient, $\vec{v}$ the flow velocity, and t the time.

Under the assumptions of steady-state conditions (i.e., time invariance) and the presumption that diffusion along the channel is negligible (i.e., flow in x-direction is much stronger than diffusion), Equation S1 simplifies to:

$$\partial_x c = \frac{D}{v_x}\left(\partial_y^2 + \partial_z^2\right) c \qquad \text{(S2)}$$

This expression can be reformulated into a dimensionless form:

$$v_x' \partial_\Phi c = \left(\partial_{y'}^2 + \partial_{z'}^2\right) c \qquad \text{(S3)}$$

with Φ being the dimensionless “diffusion coefficient”, expressed as $\Phi \equiv x\frac{D\beta}{Q}$, where x is the distance from the inlet, and β denotes the aspect ratio, which is given by $\beta = \frac{H}{W}$. The variables y' and z' are the dimensionless axes coordinates, with $y' \equiv \frac{y}{W}$ and $z' \equiv \frac{z}{H}$, respectively. The dimensionless velocity $v_x{}'$ (for specific y', z' positions) is represented as $v_x{}' \equiv v_x\frac{WH}{Q}$.

$v_x{}'$ can be determined from the incompressible Navier–Stokes equation. This equation links the fluid’s pressure p, viscosity η, and density ρ to its velocity vector $\vec{v}$ within the channel, and provides a way to compute the velocity profile in the microchannel:

$$\rho\left(\partial_t\vec{v} + \left(\vec{v} \cdot \vec{\nabla}\right)\vec{v}\right) = -\vec{\nabla}p + \eta\nabla^2\vec{v} \qquad \text{(S4)}$$

By applying the principles of time invariance and translational invariance along with the dimensionless parameters defined earlier, Equation S4 can be reformulated as:

$$\left(\partial_{y'}^2 + \partial_{z'}^2\right) v_x'(y', z') = 1 \tag{S5}$$

Equation S5 can then be solved using a series expansion method. Specifically, the velocity $v_x'(y', z', \beta)$ is given by the series:

$$v_x'(y', z', \beta) = \sum_{nm} A_{nm}(\beta) \sin(n\pi y') \sin(m\pi \frac{z'}{\beta}) \tag{S6}$$

where the coefficients $A_{nm}(\beta)$ are defined as:

$$A_{nm}(\beta) \equiv -\frac{16}{\pi^4} \frac{1}{nm\left(n^2 + \frac{m^2}{\beta^2}\right)} \delta_{(n \bmod 2),1} \delta_{(m \bmod 2),1} \tag{S7}$$

Here, δ denotes the Kronecker delta function and the variables n and m are integers used in the summation, representing the series terms for the horizontal y' and vertical z' dimensions, respectively. This formulation allows to calculate the velocity profile within the channel.

Using v_x' from Equation S6, we can now numerically integrate Equation S3 using the Crank–Nicholson method. This approach enables us to construct diffusion profiles at specific distances x within the channel, which correspond to various measurement positions, for a range of diffusion coefficients Φ (representing different R_H values). These generated profiles serve as the basis functions $B(\Phi)$.

At each measurement position, a set of these basis functions $B(\Phi)$ is generated, with each representing a distinct value of Φ (or different diffusion coefficients D). We typically use a set of 1000 logarithmically spaced Φ values, encompassing hydrodynamic radii from 0.1 nm to 1 µm. The initial concentration profile, used to compute the initial conditions for the simulations, is derived from the first fluorescence profile measured. We assume that this concentration is uniformly distributed throughout the height of the channel ($c_i(y, z) = c_i(y)$).

To ensure accurate and consistent representation across various diffusion coefficients, we normalize the basis functions $B(\Phi)$. This normalization accounts for potential variations in signal amplitude and baseline offset that may occur due to differential illumination across different images, ensuring consistent and accurate representation across the range of diffusion coefficients.

Fitting procedure. The process of fitting involves calculating the least square error between the experimental diffusion profiles and the simulated basis functions $B(\Phi)$ for each value of Φ. The fit is conducted globally across all measurement positions in the microchannel. To determine the optimal fit, we identify the two best matching basis functions $B(\Phi)$ using the sum of squared errors (SSE). These are then interpolated to ascertain the fitted diffusion coefficient Φ. The hydrodynamic radius R_H is subsequently calculated using the Stokes–Einstein relation, given by the formula:

$$R_{\mathrm{H}} = \frac{x}{Q\Phi} \beta \frac{k_B T}{6\pi\eta} \tag{S8}$$

where x is the distance from the inlet, Q is the flow rate, Φ is the dimensionless diffusion coefficient, β is the aspect ratio of the channel height to its width, k_B is the Boltzmann constant, T is the absolute temperature, and η is the fluid's viscosity.

Error calculation. The variance σ_Φ^2 is calculated using the first-order Taylor series of the least squares equation, which is given by:

$$\sigma_\Phi^2 = \frac{1}{\sum_i \left(\frac{\partial B_i(\Phi)}{\partial \Phi}\right)^2 \frac{1}{\sigma_i(\Phi)}} \tag{S9}$$

with σ_i being the noise on each point *i* of the profile. Due to the non-symmetric shape of the SSE function around the determined Φ, it is not appropriate to report errors as simple symmetric $\pm\sigma_\Phi^2$ values. To address this asymmetry, we first identify the side exhibiting the greater SSE, either $SSE(\Phi - \sigma_\Phi^2)$ or $SSE(\Phi + \sigma_\Phi^2)$, which subsequently determines the noise level. We then calculate the Φ values at which this noise level intersects with the SSE function, thus defining the error range for Φ. The two limits of the range Φ is then converted using Equation S8 to get the error range for R_H.

Analysis code. Analysis of diffusion profiles using the advection–diffusion model is done via a custom-written code in Python. The computational analysis code, developed by Q.A.E. Peter, is available on the Zenodo repository: https://doi.org/10.5281/zenodo.3881940. The current version is available on https://github.com/impact27/diffusion_device/tree/v1.0.0. For our study's specific needs, we created a dedicated branch of this original code, which includes tailored examples and instructions for analyzing traces from smMDS experiments. This branch is accessible via the GitHub repository: https://github.com/gkrainer/smMDS.

Supplementary Note 2

Statistical analysis of fitting models

To ascertain that a four-species model (i.e., one skew normal distribution and three Gaussian distributions) is indeed appropriate to describe HSA oligomer data, we applied the Bayesian Information Criterion (BIC) and the Akaike Information Criterion (AIC) to the data shown in Figure 5a. In this analysis (see **Supplementary Figure 3**), we calculated ΔBIC and ΔAIC scores as the differences between the BIC and AIC scores for each model and the model with the lowest BIC or AIC scores, respectively. Our analysis indicates that incorporating one skew normal distribution and three Gaussian distributions in the model presents the lowest ΔBIC and ΔAIC scores, substantiating that a four species model, corresponding to the equilibrium distribution of HSA monomers, dimers, trimers, and tetramers, is the most fitting. We note that for the BIC analysis, a five species model already gives a ΔBIC score of >5 and any higher-species model gives ΔBIC scores of >10. According to the criteria set forth by Raftery (1995),[2] a BIC difference of 2 to 6 suggests 'positive' evidence in favor of the model with the smaller BIC, and a difference of 6 to 10 suggests 'strong' evidence for that model. For the AIC analysis, we obtain ΔAIC scores of <2 for the five and six species models, and higher order models give scores >2. Here, according to Burnham and Anderson (2004),[3] ΔAIC scores <2 are considered to be essentially as good as the best-fitting model, whereas models with greater scores have considerably less or no support. Accordingly, the analysis based on ΔAIC scores also suggest that a four-species model is the most fitting, however, it cannot entirely rule out a five or six species model.

Supplementary Figures

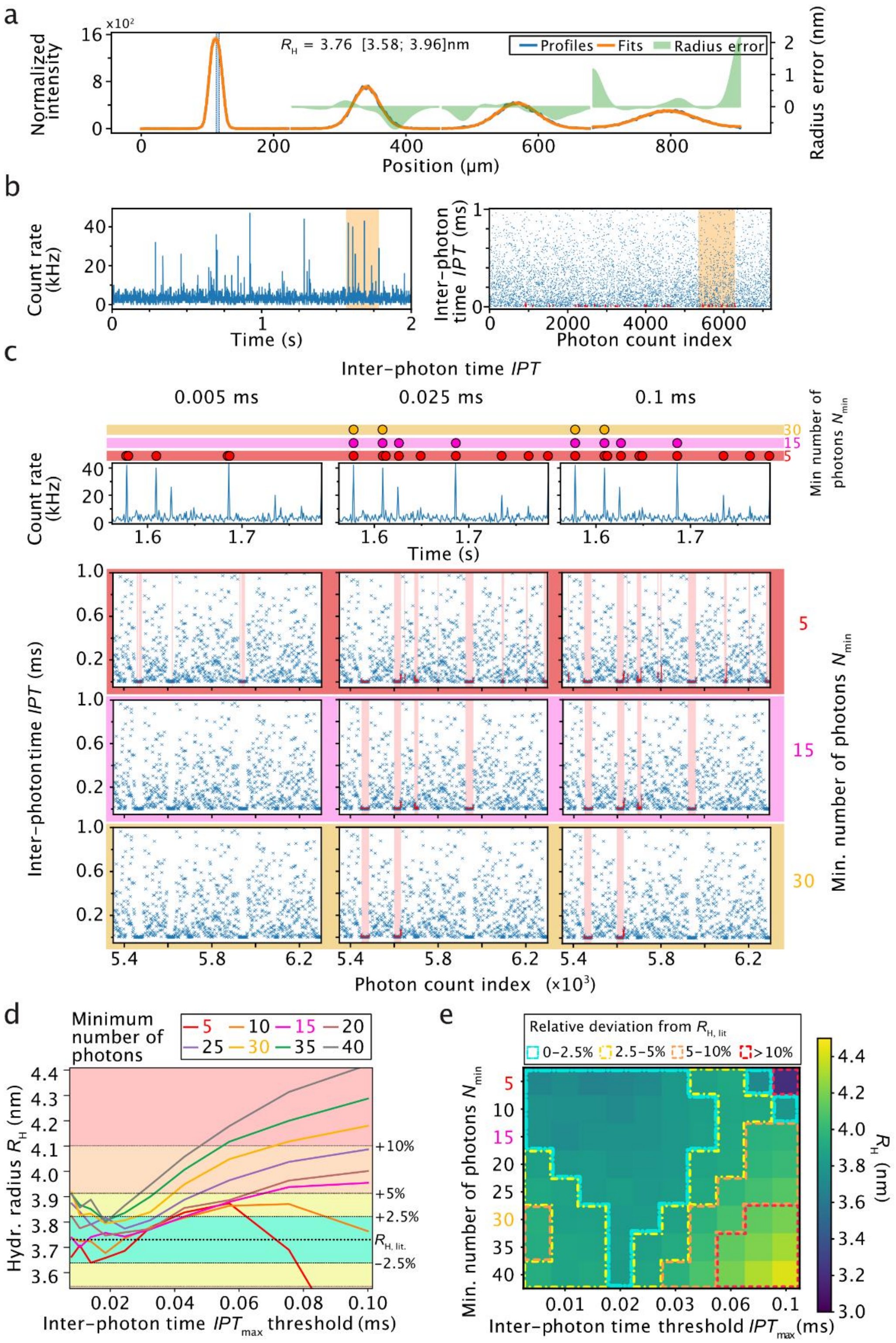

Supplementary Figure 1. Influence of burst parameter selection in smMDS experiments. (a) Diffusion profile for human serum albumin (HAS) as obtained from a step scan measurement at 20 pM protein concentration. The diffusion profile was generated from 2-second-long step scans across four channels of the microfluidic chip. Burst selection parameters used to obtain the diffusion profile were as follows: maximum inter-photon time IPT_{max} = 0.02 ms, minimum number of photons N_{min} = 15, and Lee filter 4. Diffusion profile is shown in blue, experimental fit in orange, and errors are shown as green

bands. The extracted hydrodynamic radius (R_H) [with errors] is given as an inset. **(b)** Photon-count time trace (left, 1-ms binning) and inter-photon time plot (right) at diffusion profile position 112.52 µm, as indicated with a blue line in panel a. Yellow highlighted regions in the photon-count time trace and the inter-photon time plot highlight the zoom-ins shown in panel c. Red highlighted regions in the inter-photon time plot are photon events within single-molecule bursts. **(c)** Influence of burst parameter selection on detection of single-molecule events. Shown are photon-count time traces (top panel, 1-ms binning) and inter-photon time plots (bottom panel) for different combinations of IPT_{max} (IPT_{max} = 0.005 ms, 0.025 ms, and 0.1 ms) and N_{min} thresholds (N_{min} = 5, 15, and 30) for the yellow highlighted region in panel b. Lee filter was 4 in all cases. Colored dots indicate detected single-molecule events for different N_{min} threshold values (red: N_{min} = 5, pink: N_{min} = 15, and yellow: N_{min} = 30). Red highlighted regions in the inter-photon time plots are photon events within identified single-molecule bursts. **(d)** Influence of burst parameter selection on the size determination of HSA. R_H values were extracted from diffusion profiles generated from different combinations of IPT_{max} and N_{min} values by varying IPT_{max} from 0.01–0.1 ms and N_{min} from 5–40. The dashed line indicates the average literature value for HSA ($R_{H,lit.}$ = 3.73 ± 0.40 nm)[4–7] with cyan, yellow, orange, and red bands denoting 0–2.5%, 2.5–5%, 5–10%, and >10% relative deviations from $R_{H,lit}$ values, respectively. **(e)** Two-dimensional representation of the influence of different IPT_{max} and N_{min} thresholds values on the size determination of HSA. Extracted R_H values for different combinations of IPT_{max} and N_{min} values are color coded (from R_H = 3.0 nm, blue to R_H = 4.5 nm, yellow), see color bar on the right). Regions with 0–2.5%, 2.5–5%, 5–10%, and >10% relative deviations from $R_{H,lit.}$ values are highlighted in cyan, yellow, orange, and red, respectively.

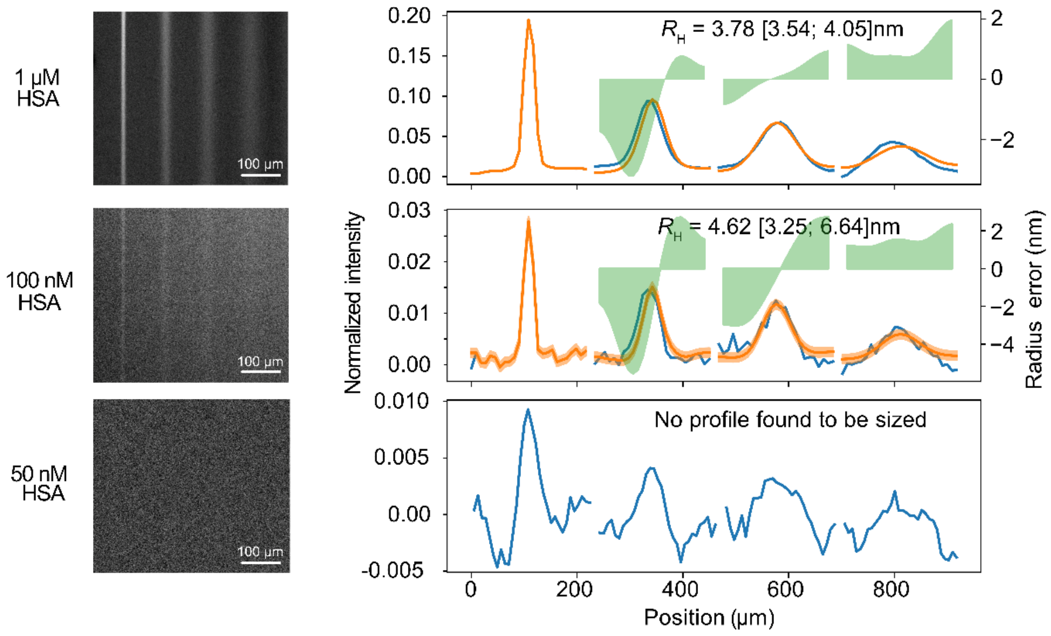

Supplementary Figure 2. Diffusional sizing of human serum albumin (HSA) by conventional microfluidic diffusional sizing (MDS) using widefield imaging. Experimental images from widefield microscopy (left panel) and diffusion profiles (right panel) of HSA taken at different protein concentrations (1 µM, 100 nM, and 50 nM HSA, from top to bottom). Experiments were performed on a custom-built epifluorescence microscope for MDS experiments as described in Schneider et al.[8] The chip was the same as used in smMDS experiments. Diffusion profiles were recorded at four different positions along the microfluidic channel. The flow rates were 100 µL/h and the buffer conditions the same as used in smMDS experiments (PBS supplemented with 0.01% Tween 20). Diffusion profiles are shown as blue lines, experimental fits as orange lines, and errors as green bands. Extracted hydrodynamic radii (R_H) [with errors] are given as insets. No fit could be obtained for the measurement at 50 nM HSA. Scale bar is 100 µm.

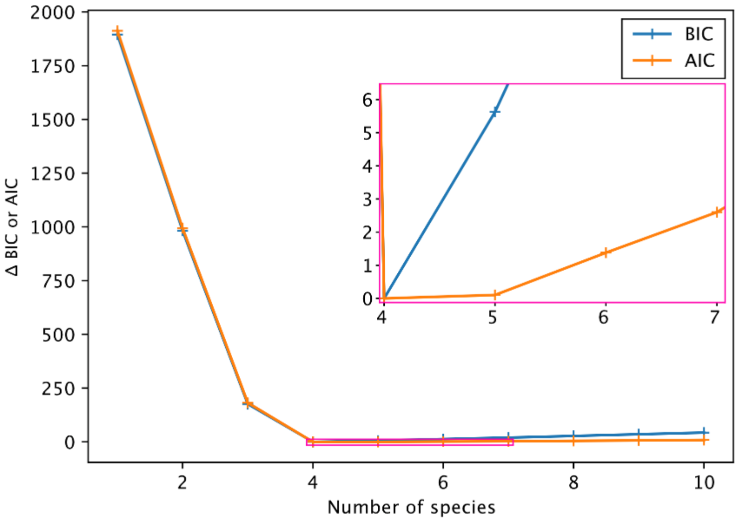

Supplementary Figure 3. Statistical analysis of various fitting models applied to the burst intensity histogram shown in Figure 5a. Displayed are the differences (ΔBIC or ΔAIC) in Bayesian Information Criterion (BIC) and Akaike Information Criterion (AIC) scores for each model relative to the model with the lowest BIC or AIC value, respectively. Each model includes one skew normal distribution and (n–1) Gaussian distributions, where n represents the total number of distributions (corresponding to the total number of species analyzed). The lowest ΔBIC and ΔAIC scores were found for the four-species model. The AIC and BIC scores were calculated from the sum of square errors (SSE) as follows, with k denoting the complexity of the model and n denoting the number of points to fit: AIC = $2k - 2\ln(\mathrm{SSE}/n)$ and BIC = $k\ln(n) - 2\ln(\mathrm{SSE}/n)$.

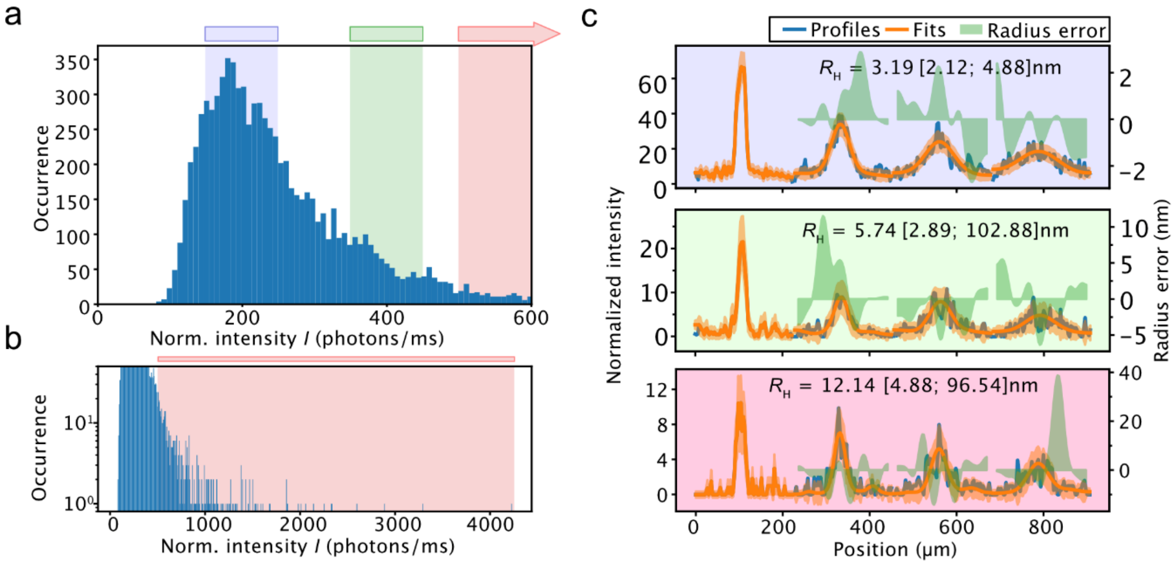

Supplementary Figure 4. Resolving α-synuclein oligomer populations by selecting defined regions in the burst intensity histogram. (a) Distribution of α-synuclein oligomer burst intensities. All bursts extracted from a smMDS step scan measurement of α-synuclein oligomers (10 pM) are displayed in a burst intensity histogram. Intensities are normalized intensities with respect to burst duration. Three regions (blue, green, and red) are selected in the burst intensity histogram. The blue and green regions span an intensity region of 100 photons/ms, starting from 150 and 350 photons/ms, respectively. The red region spans from 500 to 4230 photons/ms (indicated by the arrow). The entire red region is shown in panel b. **(b)** Burst intensity histogram in semi-log scale with red region highlighted. **(c)** Diffusion profiles generated from bursts within each of the three regions defined in panel a. The obtained diffusion

profiles were fitted to extract size information. Diffusion profiles are shown as blue lines, experimental fits as orange lines, and error as green bands. Extracted hydrodynamic radii (R_H) [with errors] are given as insets.

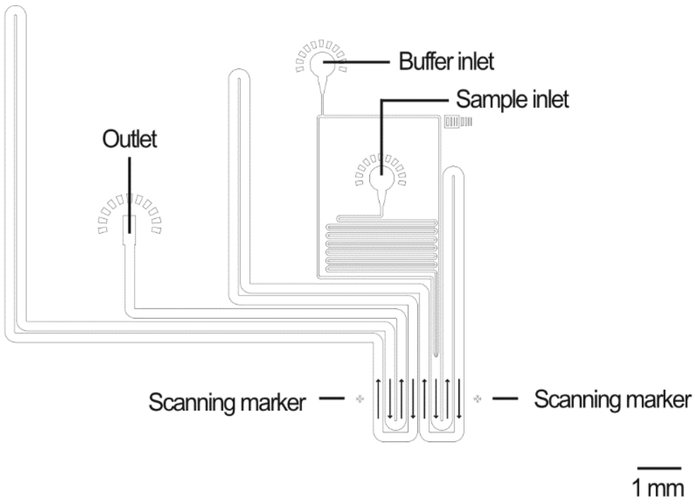

Supplementary Figure 5. Schematic of the microfluidic chip design. The most relevant features are highlighted. The arrows indicate the flow path within the channels. Scale bar is 1 mm.

Supplementary Tables

Supplementary Table 1. Extracted hydrodynamic radii of human serum albumin (HSA) as displayed in Figure 2d.

HSA concentration	Hydrodynamic radius R_H (nm)[a] Continuous scan	Step scan
100 fM		3.99 ± 0.31
1 pM		3.73 ± 0.050
10 pM		3.75 ± 0.21
20 pM	3.37 ± 0.19	3.72 ± 0.035
50 pM	3.58 ± 0.11	
100 pM	3.66 ± 0.036	
1 nM	3.71 ± 0.051	
10 nM	3.58 ± 0.037	
100 nM	3.39 ± 0.11	
1 μM	3.83 ± 0.12	

[a] Errors are reported as standard deviations.

Supplementary Table 2. Extracted hydrodynamic radii of proteins as displayed in Figure 3a.

Protein	Hydrodynamic radius R_H (nm)[a]
Alexa 488	0.60 ± 0.089
Lysozyme	1.66 ± 0.21
RNase A	2.37 ± 1.37
α-Synuclein	3.14 ± 0.37
HLA	2.86 ± 0.28
HSA	3.75 ± 0.21
Thyroglobulin	8.21 ± 0.42
α-Synuclein oligomers	7.50 ± 1.27

[a] Errors are reported as standard deviations.

Supplementary Table 3. Quantification of protein labeling.

Protein	Concentration used for labeling	Molar excess dye	Degree of labeling (DOL)	Concentration after labeling and purification
RNase A	20 μM	3x	0.90	11.8 μM
HSA	20 μM	3x	0.99	11.9 μM
Lysozyme	40 μM	3x	0.34	24.5 μM
HLA	3.12 μM	6x	0.24	734 nM
Thyroglobulin	6.45 μM	5x	0.34	2.48 μM
α-Synuclein	120 μM	3x	0.67	45.6 μM

Supplementary References

1. Peter, Q. Advances in biophysical methods for protein detection and characterisation. (University of Cambridge, 2021). doi:https://doi.org/10.17863/CAM.74582.
2. Raftery, A. E. Bayesian Model Selection in Social Research. *Sociol Methodol* **25**, 111 (1995).
3. Burnham, K. P. & Anderson, D. R. *Model Selection and Multimodel Inference: A Practical Information-Theoretic Approach*. (Springer New York, New York, NY, 2002). doi:10.1007/b97636.
4. Armstrong, J. K., Wenby, R. B., Meiselman, H. J. & Fisher, T. C. The hydrodynamic radii of macromolecules and their effect on red blood cell aggregation. *Biophys J* **87**, 4259–4270 (2004).
5. Jiang, X. *et al.* Quantitative analysis of the protein corona on FePt nanoparticles formed by transferrin binding. *J R Soc Interface* **7 Suppl 1**, (2010).
6. Akbarzadehlaleh, P., Mirzaei, M., Mashahdi-Keshtiban, M., Shamsasenjan, K. & Heydari, H. PEGylated Human Serum Albumin: Review of PEGylation, Purification and Characterization Methods. *Adv Pharm Bull* **6**, 309–317 (2016).
7. Dynamic Biosensors. List of protein hydrodynamic diameters DH. https://www.dynamic-biosensors.com/project/list-of (2022).
8. Schneider, M. M. *et al.* The Hsc70 disaggregation machinery removes monomer units directly from α-synuclein fibril ends. *Nature Communications 2021 12:1* **12**, 1–11 (2021).
