# Supplementary material for: Single-molecule digital sizing of proteins in solution
Source: Nat Commun. 2024 Sep 4;15:7740. doi: 10.1038/s41467-024-50825-9 (PMC11375031; doi:10.1038/s41467-024-50825-9)
Supplement: Supplementary file 2 — Reporting Summary [file 41467_2024_50825_MOESM2_ESM.pdf]

nature portfolio

Corresponding author(s): Georg Krainer, Tuomas P.J. Knowles

Last updated by author(s): Jul 1, 2024

Reporting Summary

Nature Portfolio wishes to improve the reproducibility of the work that we publish. This form provides structure for consistency and transparency in reporting. For further information on Nature Portfolio policies, see our Editorial Policies and the Editorial Policy Checklist.

Statistics

For all statistical analyses, confirm that the following items are present in the figure legend, table legend, main text, or Methods section.

n/a	Confirmed	
☐	☒	The exact sample size (*n*) for each experimental group/condition, given as a discrete number and unit of measurement
☐	☒	A statement on whether measurements were taken from distinct samples or whether the same sample was measured repeatedly
☒	☐	The statistical test(s) used AND whether they are one- or two-sided<br>*Only common tests should be described solely by name; describe more complex techniques in the Methods section.*
☒	☐	A description of all covariates tested
☒	☐	A description of any assumptions or corrections, such as tests of normality and adjustment for multiple comparisons
☐	☒	A full description of the statistical parameters including central tendency (e.g. means) or other basic estimates (e.g. regression coefficient) AND variation (e.g. standard deviation) or associated estimates of uncertainty (e.g. confidence intervals)
☒	☐	For null hypothesis testing, the test statistic (e.g. *F*, *t*, *r*) with confidence intervals, effect sizes, degrees of freedom and *P* value noted<br>*Give P values as exact values whenever suitable.*
☒	☐	For Bayesian analysis, information on the choice of priors and Markov chain Monte Carlo settings
☒	☐	For hierarchical and complex designs, identification of the appropriate level for tests and full reporting of outcomes
☒	☐	Estimates of effect sizes (e.g. Cohen's *d*, Pearson's *r*), indicating how they were calculated

Our web collection on statistics for biologists contains articles on many of the points above.

Software and code

Policy information about availability of computer code

Data collection	Python, version 3.6, https://www.python.org/
Data analysis	Python, version 3.6, https://www.python.org/; AutoCAD, version 2020, Autodesk, https://www.autodesk.com/; Computer code used in this article for single-molecule analysis and the analysis of diffusional sizing profiles is available on the GitHub repository: https://github.com/gkrainer/smMDS

For manuscripts utilizing custom algorithms or software that are central to the research but not yet described in published literature, software must be made available to editors and reviewers. We strongly encourage code deposition in a community repository (e.g. GitHub). See the Nature Portfolio guidelines for submitting code & software for further information.

Data

Policy information about availability of data

All manuscripts must include a data availability statement. This statement should provide the following information, where applicable:

- Accession codes, unique identifiers, or web links for publicly available datasets
- A description of any restrictions on data availability
- For clinical datasets or third party data, please ensure that the statement adheres to our policy

All the data generated in this study are available within the main text and the Supplementary Information file. Source data are provided with this paper.

Research involving human participants, their data, or biological material

Policy information about studies with human participants or human data. See also policy information about sex, gender (identity/presentation), and sexual orientation and race, ethnicity and racism.

Reporting on sex and gender	Not applicable.
Reporting on race, ethnicity, or other socially relevant groupings	Not applicable.
Population characteristics	Not applicable.
Recruitment	Not applicable.
Ethics oversight	Not applicable.

Note that full information on the approval of the study protocol must also be provided in the manuscript.

Field-specific reporting

Please select the one below that is the best fit for your research. If you are not sure, read the appropriate sections before making your selection.

[x] Life sciences [ ] Behavioural & social sciences [ ] Ecological, evolutionary & environmental sciences

For a reference copy of the document with all sections, see nature.com/documents/nr-reporting-summary-flat.pdf

Life sciences study design

All studies must disclose on these points even when the disclosure is negative.

Sample size	No statistical methods were used to pre-determine the sample size. Sample sizes were chosen according to the standards of the field (at least three replicates for each condition), which generated a sufficient number of data sets and gave sufficient statistics to describe the respective effect sizes of interest.
Data exclusions	No data were excluded from the analyses.
Replication	Reported results were replicated across multiple experiments with all replicates generating similar results. All experiment replicates were successful. Experiments were replicated or performed at least three times independently.
Randomization	Samples were not allocated into experimental groups. No randomization was required for these type of experiments. No human or animal subjects were used in the study. Randomization is not generally used in this field.
Blinding	The investigators were not blinded to the allocation during the experiments or to the outcome assessment. The data presented did not require the use of blinding. Blinding during data collection was not needed because conditions were well controlled. Blinding during analysis was not necessary as the differences between samples under different conditions were not visually apparent in the data. Finally, blinding is also not necessary because the results are quantitative and do not require subjective judgment or interpretation. Blinding is not typically used in the field.

Reporting for specific materials, systems and methods

We require information from authors about some types of materials, experimental systems and methods used in many studies. Here, indicate whether each material, system or method listed is relevant to your study. If you are not sure if a list item applies to your research, read the appropriate section before selecting a response.

Materials & experimental systems

n/a	Involved in the study	
[ ]	[x]	Antibodies
[x]	[ ]	Eukaryotic cell lines
[x]	[ ]	Palaeontology and archaeology
[x]	[ ]	Animals and other organisms
[x]	[ ]	Clinical data
[x]	[ ]	Dual use research of concern
[x]	[ ]	Plants

Methods

n/a	Involved in the study	
[x]	[ ]	ChIP-seq
[x]	[ ]	Flow cytometry
[x]	[ ]	MRI-based neuroimaging

Antibodies

Antibodies used	HLA-antibody W6/32: Mouse monoclonal anti-HLA Class I antibody [W6/32] (supplier: Abcam, cat#: ab22432, isotype: IgG2a, host species: mouse, clonality: monoclonal, clone: W6/32, concentration: 1 mg/mL)
Validation	See supplier page for validation: https://www.abcam.com/products/primary-antibodies/hla-class-i-antibody-w632-ab22432.html
