# Supplementary material for: Single-molecule digital sizing of proteins in solution
Source: Nat Commun. 2024 Sep 4;15:7740. doi: 10.1038/s41467-024-50825-9 (PMC11375031; doi:10.1038/s41467-024-50825-9)
Supplement: Supplementary file 3 — Description of Additional Supplementary Files [file 41467_2024_50825_MOESM3_ESM.pdf]

Legend for Supplementary Software

Computer code used in this article for single-molecule analysis and the analysis of diffusional sizing profiles. Code is available in a single compressed zip file ('smMDS.zip'). This folder contains two distinct scripts for the analysis of smMDS experiments: the first script (see subfolder 'SingleMoleculeAnalysis') is dedicated to the analysis of single-molecule events from step scan measurements by counting the number of bursts, or single molecules, as they pass through the confocal spot. The second script (see subfolder 'DiffusionProfileAnalysis') utilizes the data gathered by the first to calculate the hydrodynamic radius of the particles under observation. A README.txt file with instructions on how to install and use the code is provided. A description of the code's functionality is provided in the Methods section (Data analysis). An example demo dataset is also provided in the subfolder 'Example', featuring an smMDS measurement of labeled human serum albumin (HSA) at a concentration of 20 pM. The diffusion profile of this example dataset is shown in the paper in Figure 2c (top panel). The code was written in Python (version 3.6). Code is also available on the GitHub repository: https://github.com/gkrainer/smMDS. The codes are published under the GNU GENERAL PUBLIC LICENSE, Version 3, 29 June 2007; a license file ('LICENSE') is provided.
