# Supplementary figures and images for: Single-molecule digital sizing of proteins in solution
Source: Nat Commun. 2024 Sep 4;15:7740. doi: 10.1038/s41467-024-50825-9 (PMC11375031; doi:10.1038/s41467-024-50825-9)

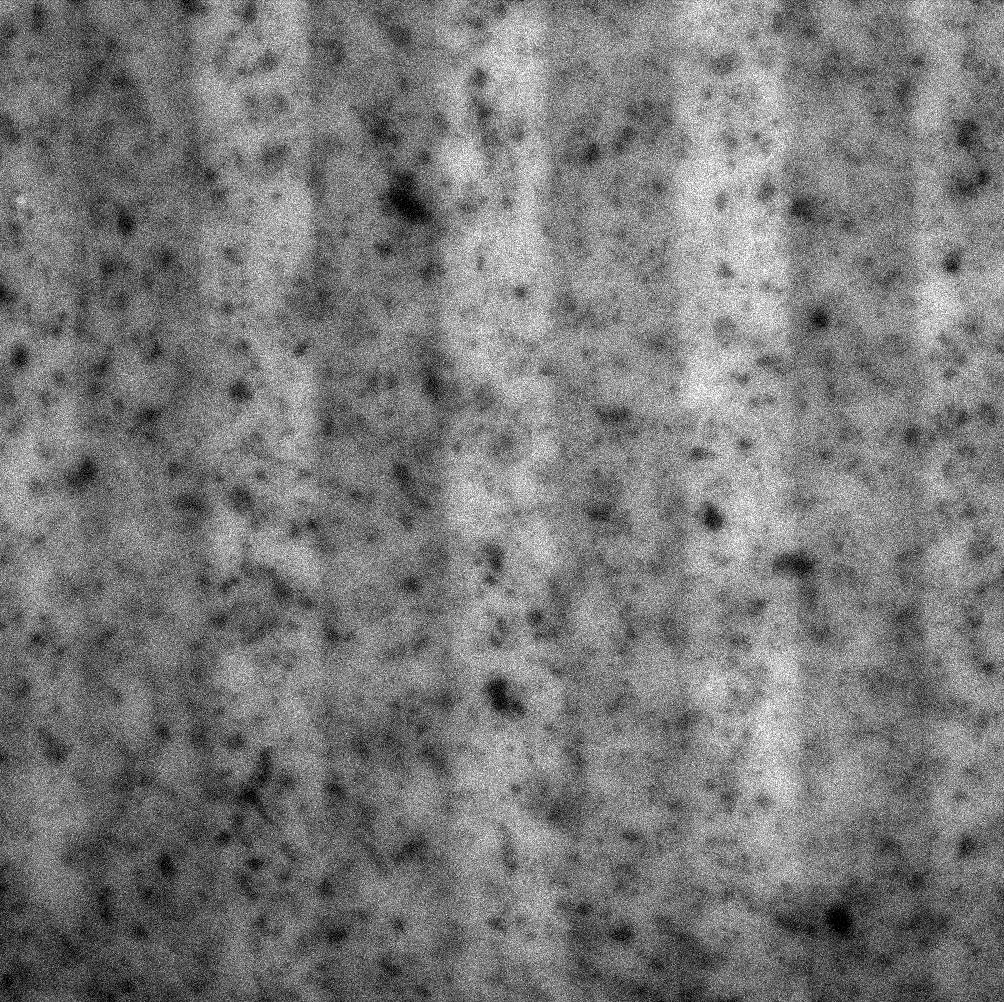

Supplement: Supplementary file 5 — Supplementary Software [file 41467_2024_50825_MOESM5_ESM.zip › smMDS/smMDS/DiffusionProfileAnalysis/Samples/SampleData/UVbg.tif]

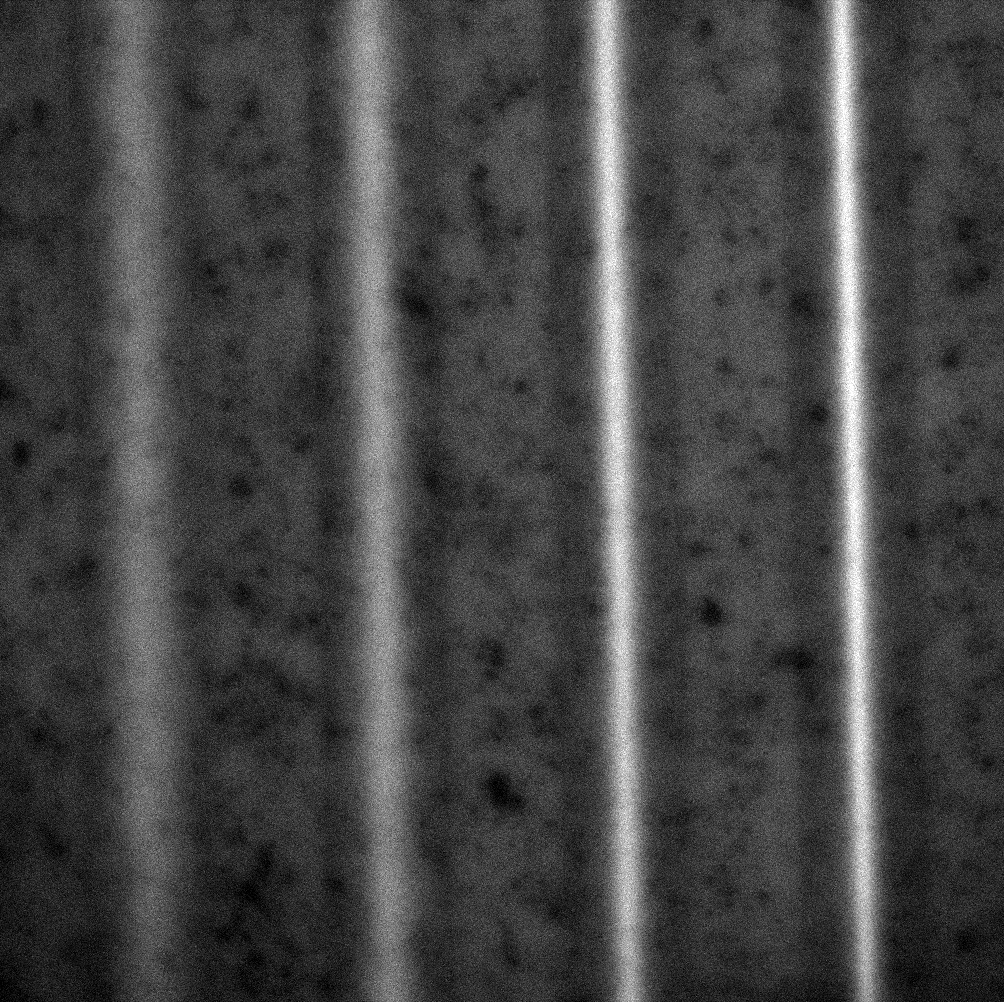

Supplement: Supplementary file 5 — Supplementary Software [file 41467_2024_50825_MOESM5_ESM.zip › smMDS/smMDS/DiffusionProfileAnalysis/Samples/SampleData/UVim300ulph.tif]

$r = 3.69 [3.51; 3.88] \text{ nm}$ , LSE = 0.599, pixel = 2.961  $\mu\text{m}$

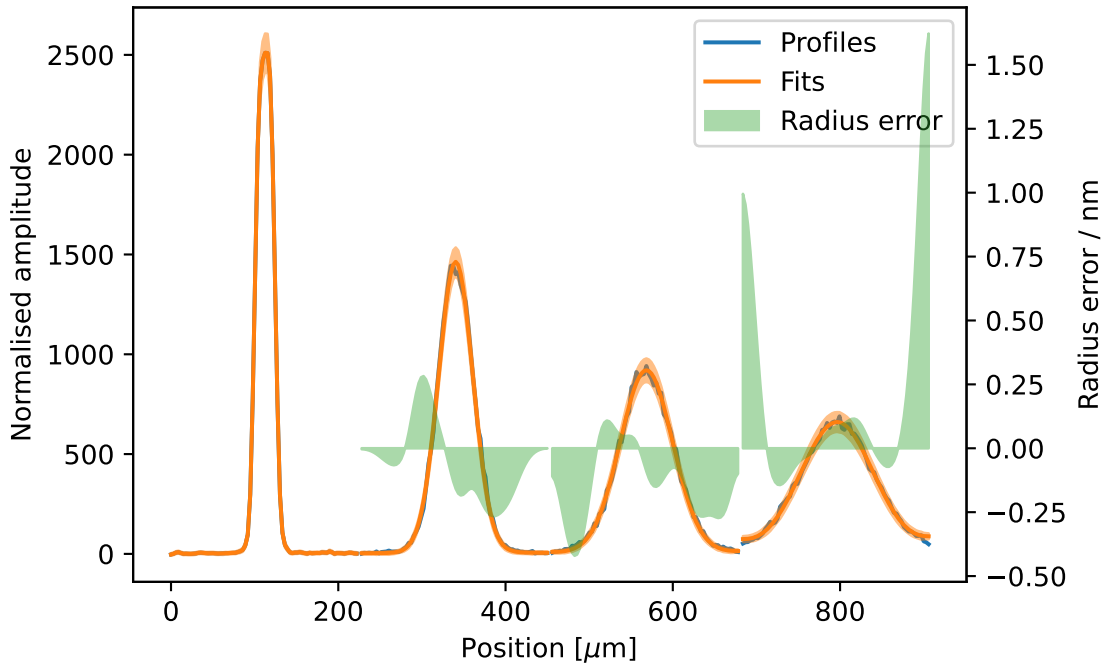

Supplement: Supplementary file 5 — Supplementary Software [file 41467_2024_50825_MOESM5_ESM.zip › smMDS/smMDS/Example/ExpectedResults/DiffusionProfileAnalysis/Alldata_fig.pdf]

# Walls

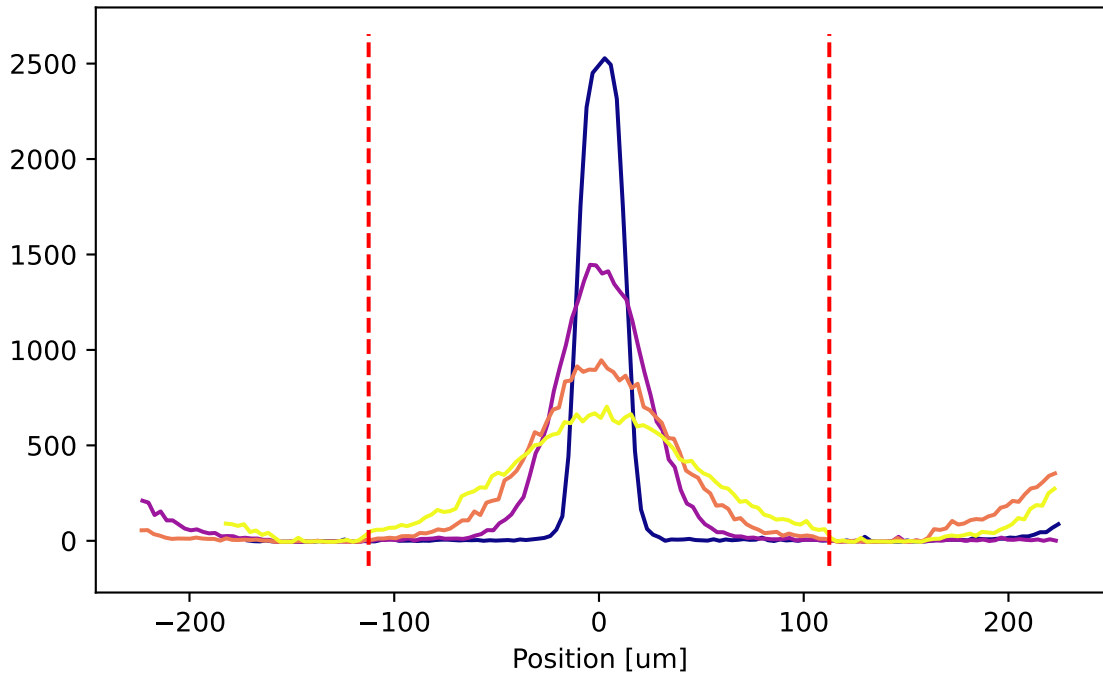

Supplement: Supplementary file 5 — Supplementary Software [file 41467_2024_50825_MOESM5_ESM.zip › smMDS/smMDS/Example/ExpectedResults/DiffusionProfileAnalysis/Alldata_walls.pdf]
